# Supplementary material for: Pollution Levels and Risk Assessment of Heavy Metals in the Soil of a Landfill Site: A Case Study in Lhasa, Tibet
Source: Int J Environ Res Public Health. 2022 Aug 27;19(17):10704. doi: 10.3390/ijerph191710704 (PMC9517830; doi:10.3390/ijerph191710704)
Supplement: Supplementary file 1 [file ijerph-19-10704-s001.zip › ijerph-1887062-supplementary.pdf]

Table S1. Ecological risk analysis standard of potential ecological risk assessment.

E_r^i	Pollution level	RI	Pollution level
$E_r^i < 40$	Low ecological hazard	RI < 150	Low ecological risk
$40 \le E_r^i < 80$	Moderate ecological hazard	150 ≤ RI < 300	Moderate ecological risk
$80 \le E_r^i < 160$	Considerable ecological hazard	300 ≤ RI < 600	Considerable ecological risk
$160 \le E_r^i < 320$	high ecological damage	RI ≥ 600	high ecological risk
$E_r^i \ge 320$	very high ecological hazard	-	-

Note：- stands for not acquirable

Table S2. Exposure factors for risk assessment models.

Exposure parameters	The parameter name	Reference (adult)	unit	The data source
OSIRa	Daily soil intake	100	mg/d	Technical guidelines for risk assessment of contaminated sites (2014)
ABSo	Absorption efficiency factor by ingestion	1	—	
SAF	Reference dose distribution coefficient	0.2	—	
SAEa	Exposed skin surface area	1.614	cm^2	
SSARa	Soil adhesion coefficient of skin surface	0.2	mg/cm^2	
Ev	Frequency of daily skin contact	1	times/d	
ABSd	Skin contact absorption efficiency factor	As:0.03; Cd:0.001	—	
PM10	Particulate matter content	0.15	mg/m^3	
DAIRa	Daily air respiration	14.5	m^3/d	
PIAF	Soil particle retention ratio	0.75	—	
fspo	The proportion of particulate matter from soil in indoor air	0.5	—	
fspi	The proportion of particulate matter from soil in outdoor air	0.8	—	
BWa	Weight	64	Kg	National Health Commission of the people′s Republic of China, 2019., Wang et al,2008., Technical guidelines for risk assessment of contaminated sites (2014).
EFOa	Outdoor exposure frequency	62.5	d/a	
EFIa	Indoor exposure frequency	187.5	d/a	
EDa	Exposure period	25	a	
EFa	Exposure frequency	250	d/a	
ATca	Average duration of carcinogenic effects	26280	d	
ATnc	Average duration of non-carcinogenic effects	9125	d	

Table S3. Reference doses (RfDs) of heavy metal elements in topsoil.

Heavy metal	$SF_{o\ (mg/kg \cdot d)}^{-1}$	$SF_{i\ (mg/kg \cdot d)}^{-1}$	$SF_{d\ (mg/kg \cdot d)}^{-1}$	$RfD_{i\ (mg/kg \cdot d)}$	$RfD_{o\ (mg/kg \cdot d)}$	$RfD_{d\ (mg/kg \cdot d)}$
Cd	7.5	7.94	300	2.27E-06	1.00E-03	2.50E-05
AS	1.5	18.9	1.5	3.40E-06	3.00E-04	3.00E-04
Cr	--	--	--	2.27E-05	3.00E-03	7.50E-05
Cu	--	--	--	9.06E-05	4.00E-02	4.00E-02
Zn	--	--	--	2.83E-01	3.00E-01	3.00E-01
Ni	--	--	--	2.04E-05	2.00E-02	8.00E-04
Pb	--	--	--	3.31E-03	3.50E-03	3.50E-03
Hg	--	--	--	6.80E-05	3.00E-04	0.000021

(Technical guidelines for risk assessment of contaminated sites; USEPA Integrated Risk Information System; Duan et al.,2009, 2011)
